# Nuclear Quantum Effects on the Organic Bifurcation Reaction in Microsolvated Water Clusters: Ring‐Polymer Molecular Dynamics Calculations Using an Explicit Solvation Model

**DOI:** 10.1002/jcc.70458

**Published:** 2026-07-06

**Authors:** Shoto Nakagawa, Hayato Matsubuchi, Haruki Ota, Toshiyuki Takayanagi, Tatsuhiro Murakami

**Affiliations:** ^1^ Department of Chemistry Saitama University Saitama Japan; ^2^ Department of Applied Chemistry for Environment, Graduate School of Urban Environmental Sciences Tokyo Metropolitan University Tokyo Japan

**Keywords:** explicit solvation model, GFN2‐xTB, nuclear quantum effect, post‐transition state bifurcation, ring‐polymer molecular dynamics

## Abstract

Solvent environments often reshape reaction mechanisms compared to those obtained in the gas phase or in nonpolar solvents. Recently, explicit solvation models—where individual solvent molecules are treated—have been increasingly employed in simulations of organic reactions to capture the dynamic influence of solvent motions. In aqueous systems, the incorporation of nuclear quantum effects (NQEs) is particularly crucial for accurately describing both structural and dynamical features. Here, we investigate the bifurcation reaction between 2‐aminoacrolein and 1,3‐butadiene in microsolvated (H_2_O)_*n*_ clusters (*n* = 5, 15, 45) using ring‐polymer molecular dynamics (RPMD), and compare the results with our previous classical molecular dynamics (classical MD) simulations. The branching fractions obtained from RPMD trajectories exhibit an increased tendency toward the minor (4 + 2) product pathway—equivalently, a lower fraction of the dominant (4 + 3) channel—compared with classical MD, owing to zero‐point energy contributions distributed across all vibrational modes of the system. Moreover, RPMD reveals significantly accelerated proton‐transfer events, indicating that nuclear quantum effects, including zero‐point energy and proton delocalization, contribute substantially even at 300 K. These findings demonstrate that reliable prediction of aqueous branching behavior and proton‐transfer kinetics requires both explicit solvation and rigorous inclusion of NQEs.

## Graphical abstract

Ring‐polymer molecular dynamics in explicit water clusters shows that nuclear quantum effects steer an organic bifurcation reaction—enhancing the minor (4 + 2) channel and accelerating water‐mediated proton transfer—proving that accurate aqueous reaction dynamics require both explicit solvation and nuclear quantum effects.

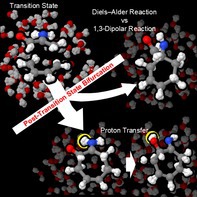

## 1 Introduction

When organic reactions proceed in aqueous environments, their mechanisms and product selectivities often diverge substantially from those obtained in the gas phase or in nonpolar solvents. This divergence arises because polar solvent molecules can directly reshape the underlying potential energy surface through electrostatic interactions with the solute. To evaluate the influence of polar solvation on the reactive energy landscape, implicit solvation approaches, such as polarizable continuum models (PCMs), are commonly applied in quantum chemical calculations [1, 2, 3, 4]. Although these implicit solvation models are very useful for characterizing key features of the potential energy landscape, such as transition‐state geometries and activation barriers, they do not provide detailed atomistic information on solvent dynamics during reactions, including non‐equilibrium energy transfer between solute and solvent. To fully capture these dynamical solvent effects, it is necessary to employ explicit solvation models in which individual solvent molecules are explicitly included in simulations of organic reactions in solution [5, 6, 7].

Another crucial aspect of organic reactions in aqueous solution is the role of nuclear quantum effects (NQEs) in reaction dynamics. Although many prior dynamical studies have employed explicit solvation models, the motions of water molecules have typically been treated within a classical molecular dynamics (MD) framework [8, 9]. It should be emphasized that a purely classical description of nuclear motion cannot fully capture the quantum mechanical nature of hydrogen atoms in aqueous environments. Even at room temperature, zero‐point motion and the associated quantum delocalization of light nuclei significantly influence hydrogen‐bond strengths, O—H bond‐length distributions, and the stability of charge‐separated structures [10, 11, 12, 13]. These quantum effects can influence proton‐shuttling pathways and modify effective free‐energy barriers. NQEs are ubiquitous in water and aqueous systems, affecting thermodynamic response functions, diffusion behavior, and isotope‐dependent properties, and are now recognized as a systematic source of deviations from classical predictions [14, 15, 16, 17, 18, 19]. A rigorous finite‐temperature approach to incorporating NQEs is provided by path‐integral–based simulations, in which each quantum nucleus is represented by a classical ring polymer, enabling efficient sampling of quantum fluctuations from molecular clusters to extended systems. For example, ab initio path‐integral molecular dynamics (PIMD) has demonstrated that the elastic constants of ice VII change significantly under high pressure as a consequence of NQEs [18]. Studies comparing light and heavy water have also revealed systematic isotope effects on structural parameters and evaporation energies [19]. From the standpoint of ring‐polymer molecular dynamics (RPMD), an approximate real‐time extension of path‐integral theory [20, 21, 22, 23, 24, 25, 26, 27, 28, 29, 30, 31, 32, 33, 34, 35, 36, 37, 38, 39, 40], NQEs have been demonstrated to enhance hydrogen‐bond network rearrangements and accelerate proton reorganization in hydrogen‐bonded systems [24]. Collectively, these findings highlight the necessity of incorporating NQEs for a reliable description of organic reactions in explicit aqueous environments.

**FIGURE 1:**
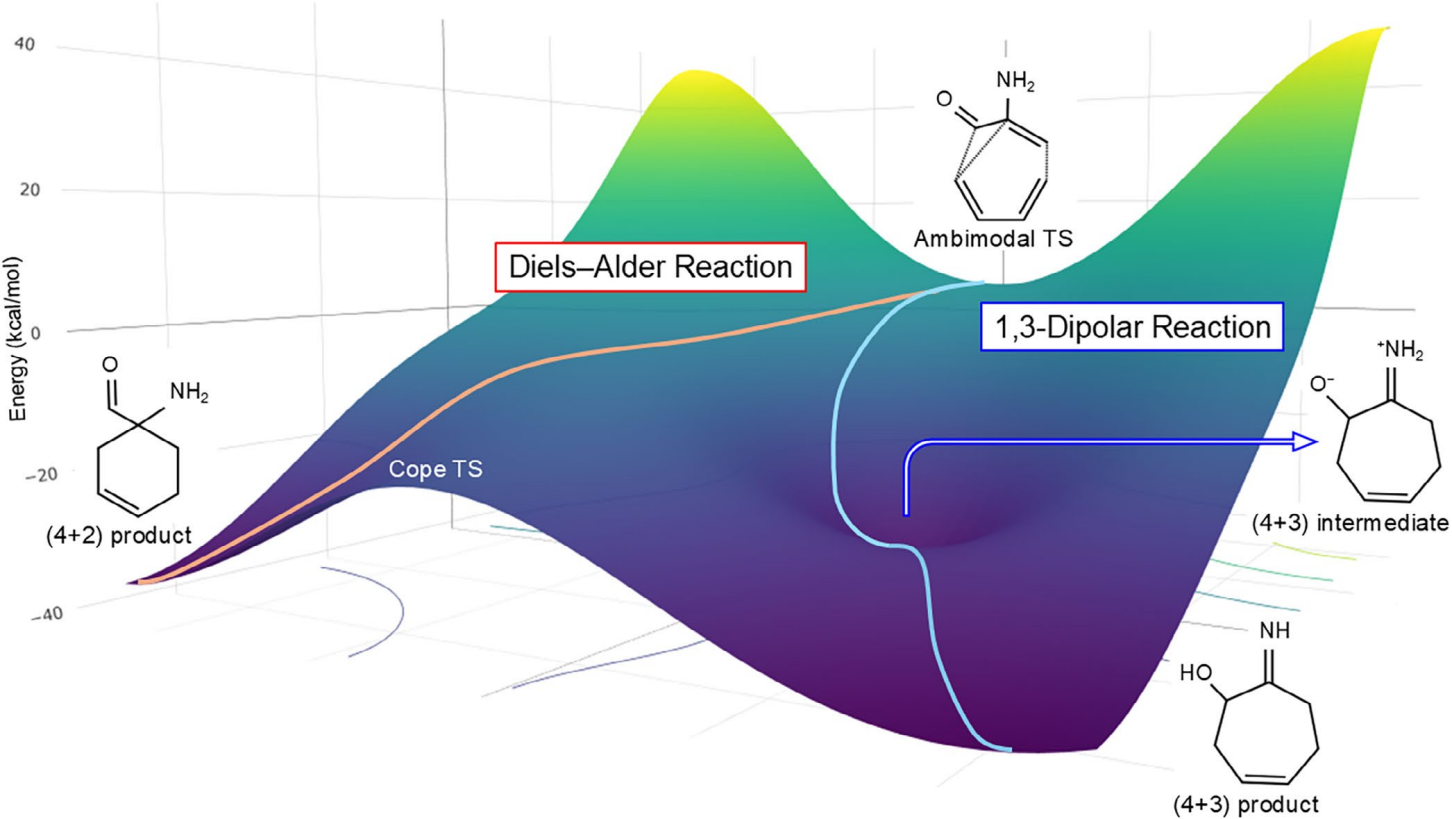
Two‐dimensional schematic potential energy surface and energy diagram for the PTSB reaction between 2‐aminoacrolein and 1,3‐butadiene, including the molecular structures of the ambimodal TS, the (4 + 2) product, the (4 + 3) intermediate, and the (4 + 3) product. The orange and light blue curves represent the pathways leading to the (4 + 2) and (4 + 3) products, respectively.

In this work, we investigate the cyclization reaction between 2‐aminoacrolein and 1,3‐butadiene in aqueous environments, focusing specifically on the role of NQEs in the reaction dynamics using the RPMD method combined with a water‐cluster model. Previous theoretical studies have established that this reaction exhibits post‐transition‐state bifurcation (PTSB), where two cyclization pathways—leading to the Diels–Alder (4 + 2) six‐membered and dipolar (4 + 3) seven‐membered cycloaddition products—bifurcate from a single ambimodal transition state (TS) structure [41, 42, 43]. The reaction scheme is depicted in Figure 1. Houk and co‐workers conducted foundational computational studies of this system in both the gas phase and aqueous solution using a PCM‐based implicit solvation model. Their results demonstrated that polar solvents significantly affect bifurcation dynamics by stabilizing the charge‐separated dipolar character in the Cope TS region of the potential energy surface [41]. As a result, the branching fractions leading to the (4 + 2) six‐membered product and to the (4 + 3) seven‐membered product differ substantially between gas‐phase and aqueous environments. Motivated by this study, our group previously performed reaction dynamics simulations using explicit microsolvation models containing 5–45 water molecules and demonstrated that microsolvation reshapes the potential energy surface, influencing the bifurcation behavior [43]. However, those simulations were carried out using classical MD and thus did not incorporate NQEs. Incorporating NQEs is, however, expected to be important for the bifurcation step itself, and not only for processes occurring after it. Because the partitioning between the (4 + 2) and (4 + 3) channels is governed by the non‐statistical dynamics in the region immediately following the ambimodal TS, the zero‐point energy distributed over all vibrational modes of the system, together with the quantum delocalization of the light nuclei, can vary the effective forces experienced by the bifurcating trajectories and thereby redistribute them between the two products. Such effects have been shown to shift PTSB branching ratios by several percent in gas‐phase and implicit‐solvent studies, typically enhancing the minor channel [42], and they are absent by construction from a classical‐MD description. Clarifying how NQEs modify the branching behavior under explicit aqueous solvation is therefore the first objective of the present study. A second, distinct aspect of this PTSB reaction is that formation of the (4 + 3) product involves a subsequent proton‐transfer step from the (4 + 3) intermediate to the final product, as illustrated in Figure 1. Two distinct proton‐transfer pathways are possible: a direct intramolecular transfer from the NH_2_^+^ group to the O^−^ moiety, and an indirect pathway mediated by surrounding water molecules. Although this step occurs after the branching event and therefore does not itself change the branching fraction, it is strongly affected by NQEs and could not be characterized with classical MD; in our previous study it was not examined in detail. In the present work, we employ RPMD to quantitatively evaluate the influence of NQEs both on the bifurcation branching and on the subsequent proton‐transfer pathways, which allows us to achieve a more comprehensive understanding of their contribution to the overall reaction dynamics.

## 2 Method

**FIGURE 2:**
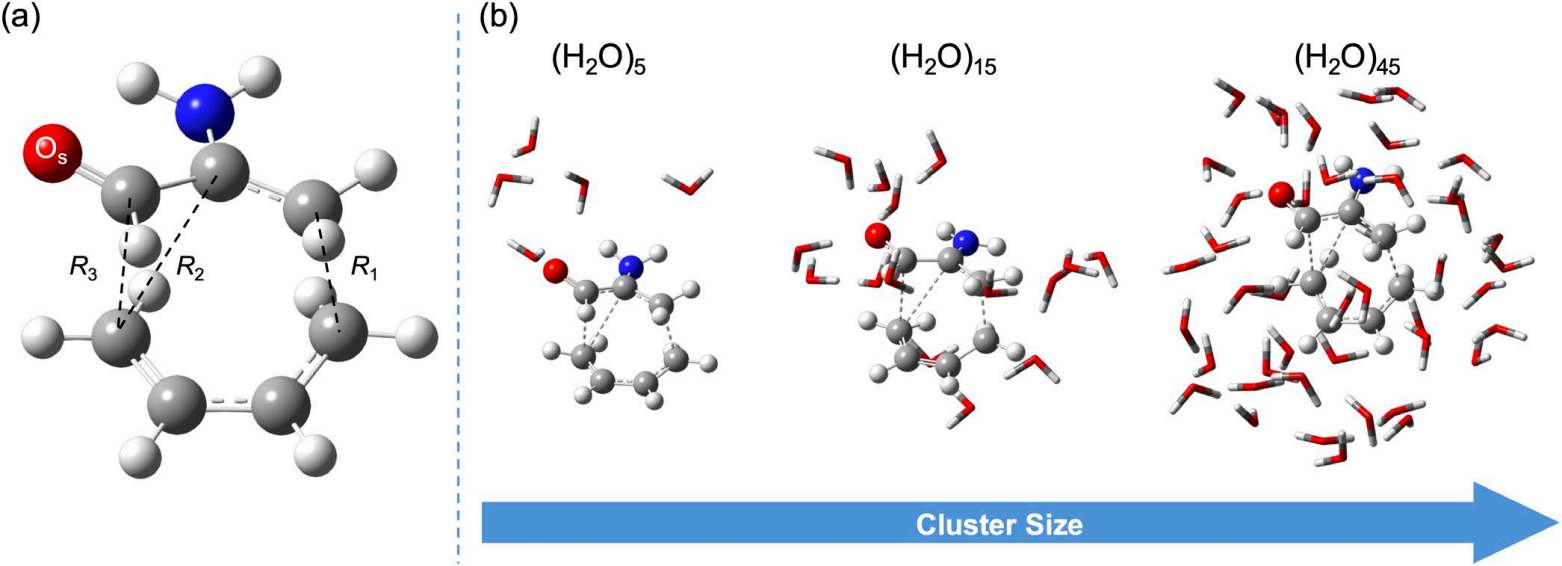
(a) Ambimodal TS structure in the gas phase with the three key C—C internuclear distances, *R*_1_, *R*_2_, and *R*_3_. The O_s_ denotes the oxygen atom in 2‐aminoacrolein. (b) Representative ambimodal TS structures solvated by (H_2_O)_5_ (left), (H_2_O)_15_ (middle), and (H_2_O)_45_ (right) clusters.

In this study, we conducted dynamical simulations for the cluster reaction systems consisting of 2‐aminoacrolein and 1,3‐butadiene with three sizes of water molecules, (H_2_O)_5_, (H_2_O)_15_, and (H_2_O)_45_, as illustrated in Figure 2. Figure 2a defines the key internuclear distances *R*_1_, *R*_2_, and *R*_3_, used in the sampling procedure, while Figure 2b illustrates representative ambimodal TS structures of the corresponding solvated clusters. The setup follows our previous classical MD study [42]. All molecular dynamics simulations were performed using the PIMD code developed by Shiga [44]. We have slightly modified the PIMD code to interface the GFN2‐xTB code [45, 46, 47]. As for reactive potential energy surfaces or reactive force fields for the whole reaction system, we employed the parameter‐optimized semiempirical GFN2‐xTB model, which reproduces DFT‐B3LYP(D3)/6‐31+G(d,p) energies and gradients in common with our previous works [42, 43]. This model was constructed by adjusting only a small subset of parameters associated with the reacting framework, while the surrounding water molecules are described with the standard GFN2‐xTB parameters, so that the same parameter set is directly transferable to the solvated clusters. To confirm that the model remains accurate in the solvated systems, we recomputed 17 representative IRCs for the (H_2_O)_15_ cluster–spanning the ambimodal TS region, the (4 + 2)/(4 + 3) branching region, the zwitterionic (4 + 3) intermediate, and the proton‐transfer barrier region at the B3LYP‐D3/6‐31+G(d,p) level using ORCA 6.1.1 [48]. The parameter‐optimized model reproduces the DFT relative‐energy profiles with mean RMSDs of 1.9–3.3 kcal/mol across these regions, substantially smaller than those of the default GFN2‐xTB model, which exceed 13 kcal/mol in the ambimodal‐TS and branching regions (see Figures S1 and S2 of Section S1). The dynamics calculations were initiated from the structures near the ambimodal TS region. These initial coordinates and momenta were obtained from constant‐temperature molecular dynamics simulations at *T* = 300 K, using three appropriate harmonic bias potential energy terms applied to the *R*_1_, *R*_2_, and *R*_3_ internuclear distances shown in Figure 2a. The centers of the bias terms were set at 2.06, 2.81, and 2.59 Å for *R*_1_, *R*_2_, and *R*_3_, respectively, corresponding to the internuclear distances optimized at the ambimodal TS structure in the gas‐phase with the parameter‐optimized GFN2‐xTB method [42]. Temperature control was maintained using a Nosé–Hoover chain thermostat. After the initial configurations were selected, the bias potentials were removed and the trajectories were propagated forward in time. We note that *R*_1_, *R*_2_, and *R*_3_ are intramolecular C–C distances of the reacting solute framework; the ambimodal TS distances optimized in the presence of the (H_2_O)_15_ cluster (*R*_1_ = 2.25, *R*_2_ = 2.91, *R*_3_ = 2.57 Å; Table S1 of Section S1) deviate from these gas‐phase reference values by at most ~0.2 Å. The gas‐phase distances, used as common sampling centers for all cluster sizes, therefore place the initial configurations within the relevant ambimodal TS region and allow a consistent comparison of solvent effects. For each cluster size, 1000 trajectories were propagated with a time step of 0.1 fs up to a total simulation time of 500 fs. Both PIMD sampling and RPMD simulations employed 32 beads for all reaction systems, which was verified to be sufficient to accurately account for NQEs at *T* = 300 K. Furthermore, several intrinsic reaction coordinate (IRC) calculations were carried out at the parameter‐optimized GFN2‐xTB level using the GRRM package [49, 50, 51].

## 3 Results and Discussion

**FIGURE 3:**
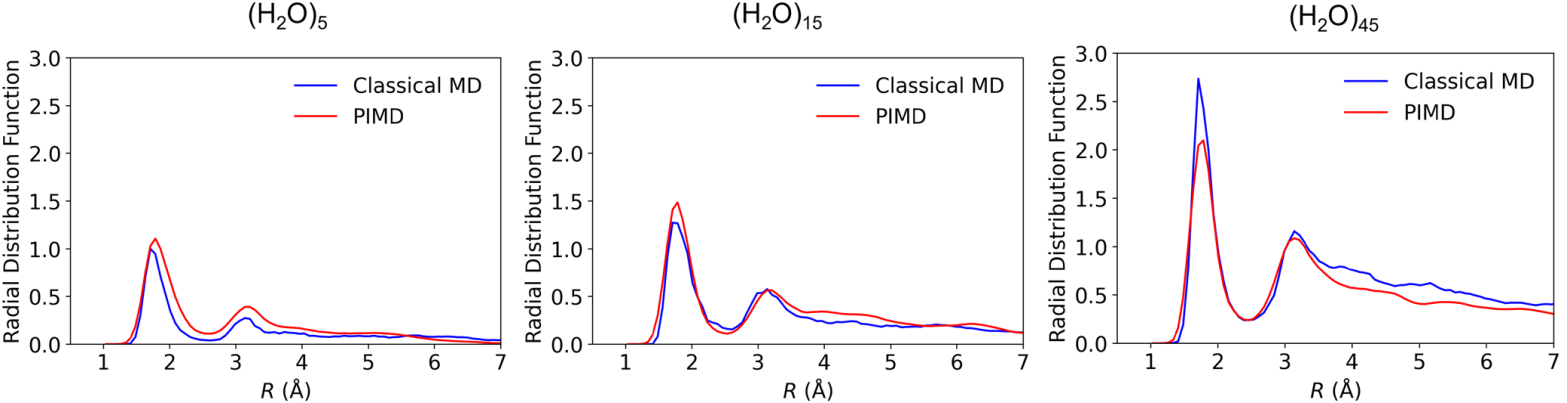
Radial distribution functions between the oxygen atom in 2‐aminoacrolein and the hydrogen atoms of water molecules obtained from classical MD (blue lines) and PIMD (red lines) for (H_2_O)_5_ (left), (H_2_O)_15_ (middle), and (H_2_O)_45_ (right) clusters.

To compare the water distributions in the initial configurations used for real‐time dynamics, obtained from the PIMD scheme and the classical approach, Figure 3 shows the radial distribution functions for each cluster system as a function of the distance (*R*) between the oxygen atom (O_s_) of 2‐aminoacrolein (see Figure 2a) and the hydrogen atoms of the surrounding water molecules. For each cluster system, the first peaks appear at approximately 1.8 Å, indicating that the interactions between the O_s_ and the hydrogen atoms of water in the first solvation shell are comparable to typical hydrogen‐bond distances observed between water molecules, consistent with the second peak of the oxygen–hydrogen radial distribution function in bulk water [19, 52]. The peak broadening depicted in Figure 3 due to NQEs is more prominent in smaller clusters, indicating that in the (H_2_O)_5_ system the hydrogen atoms of water more readily interact with the O_s_ as a consequence of enhanced quantum fluctuation. In contrast, as the number of water molecules increases, the broadening of the first peak in the PIMD results becomes less significant, while the corresponding classical peak height increases. To quantify the differences in solvation between classical MD and PIMD, we evaluated the average number of water hydrogen atoms within the first solvation shell, obtained by integrating the O_s_–H radial distribution function up to a common first‐minimum cutoff of 2.6 Å applied to both methods (see Section S2). As summarized in Table 1, the PIMD first‐shell count exceeds the classical one for the (H_2_O)_5_ and (H_2_O)_15_ clusters, whereas it becomes slightly smaller for (H_2_O)_45_. Nuclear quantum effects are known to have competing influences on hydrogen bonding [10, 11, 12, 13]: the present trend indicates that quantum delocalization of the light hydrogen atoms enhances the O_s_–water contact in the smaller clusters, raising the first‐shell count, while in the more bulk‐like (H_2_O)_45_ environment, where the first solvation shell is already well established, the delocalization causes the first solvation shell to broaden slightly, leading to a decrease in the first‐shell population.

**TABLE 1:** Average number of hydrogen atoms in the first solvation shell surrounding the oxygen atom (O_s_) of 2‐aminoacrolein, obtained from classical MD and PIMD simulations for the (H_2_O)_5_, (H_2_O)_15_, and (H_2_O)_45_ cluster systems.

	Classical MD	PIMD
(H_2_O)_5_	1.09	1.70
(H_2_O)_15_	1.89	2.04
(H_2_O)_45_	3.09	2.90

**FIGURE 4:**
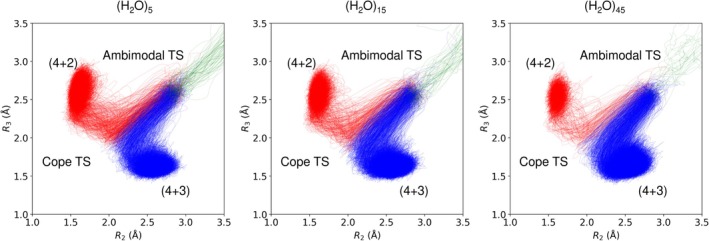
Two‐dimensional centroid trajectory maps plotted as functions of (*R*_2_, *R*_3_) obtained from RPMD simulation for (H_2_O)_5_ (left), (H_2_O)_15_ (middle), and (H_2_O)_45_ (right) clusters. The red and blue lines represent the trajectories leading to the (4 + 2) and (4 + 3) products, respectively. The green lines correspond to the trajectories returning to the reactants.

Here, we discuss the reaction dynamics. Figure 4 shows the 1000 trajectories obtained from the present RPMD simulations at *T* = 300 K, projected onto the (*R*_2_, *R*_3_) plane (see Figure 2a). In this figure, trajectories leading to the (4 + 2) six‐membered product are depicted in red, whereas those forming the (4 + 3) seven‐membered product are shown in blue; the other trajectories that return to the reactant region are displayed in green. Consistent with our previous classical MD results [43], the formation of the seven‐membered ring becomes increasingly favored as the number of water molecules increases. This solvent dependence reflects how the relevant transition‐state structures respond to microsolvation. Whereas the ambimodal TS geometry is only weakly perturbed by solvation—the C—C bond distances *R*_1_, *R*_2_, and *R*_3_ differ by at most ~0.2 Å between the gas phase and the (H_2_O)_15_ cluster (Table S1)—the Cope TS, where the solute acquires pronounced charge‐separated character, is far more sensitive to the surrounding water, as emphasized in our previous study [43]. The preferential stabilization of this charge‐separated Cope TS region by polar water is what shifts the bifurcation toward the (4 + 3) channel as the cluster size increases. Table 2 summarizes the branching fractions for the (4 + 2) and (4 + 3) forms, as well as the number of trajectories leading to each final structure in the reaction between 2‐aminoacrolein and 1,3‐butadiene. Note that, for the (4 + 3) channel, both the zwitterionic (4 + 3) intermediate and final (4 + 3) product structures are included, where we ignore the proton‐transfer process at this stage. Comparison of the RPMD and classical MD simulations revealed that RPMD yields a slightly lower fraction of the (4 + 3) product. Under the present solvated conditions, the (4 + 3) seven‐membered channel is the dominant (major) pathway and the (4 + 2) six‐membered channel is the minor pathway; the lower (4 + 3) fraction obtained with RPMD therefore corresponds to an increased population of the minor (4 + 2) channel. More specifically, the RPMD and classical MD branching fractions differ by 3%–9% across the three clusters, larger than the corresponding 68% confidence intervals (Table 2) and therefore statistically significant. A plausible explanation for this discrepancy is the inclusion of zero‐point energy (ZPE) in RPMD, since ZPE contributes to all vibrational modes of every molecule in the system. Consequently, incorporation of ZPE can promote formation of products that deviate from the dominant IRC pathway. Previous studies in both gas‐phase and PCM environments have similarly reported that branching ratios in PTSB‐type reactions can shift by several percent due to NQEs, often enhancing the population of the minor pathway [42].

**TABLE 2:** Branching fractions leading to the (4 + 2) and (4 + 3) species obtained from classical MD and RPMD simulations for the (H_2_O)_5_, (H_2_O)_15_, and (H_2_O)_45_ cluster systems.

	Classical MD [43]		RPMD	
	(4 + 2)	(4 + 3)	(4 + 2)	(4 + 3)
(H_2_O)_5_	0.387 ± 0.016 (348)	0.613 ± 0.016 (552)	0.419 ± 0.016 (391)	0.581 ± 0.016 (543)
(H_2_O)_15_	0.233 ± 0.014 (221)	0.767 ± 0.014 (727)	0.274 ± 0.014 (263)	0.726 ± 0.014 (696)
(H_2_O)_45_	0.044 ± 0.007 (44)	0.956 ± 0.007 (948)	0.134 ± 0.011 (132)	0.866 ± 0.011 (853)

*Note:* Each fraction is given with its 68% confidence interval, estimated from binomial counting statistics as $\sqrt{p\left(1-p\right)/N}$, where p is the fraction and N is the number of trajectories that formed a product. The numbers in parentheses represent the number of trajectories that result in formation of the (4 + 2) or (4 + 3) species. For each cluster and method, these counts sum to fewer than the 1000 propagated trajectories because the remaining trajectories return to the reactant region.

**FIGURE 5:**
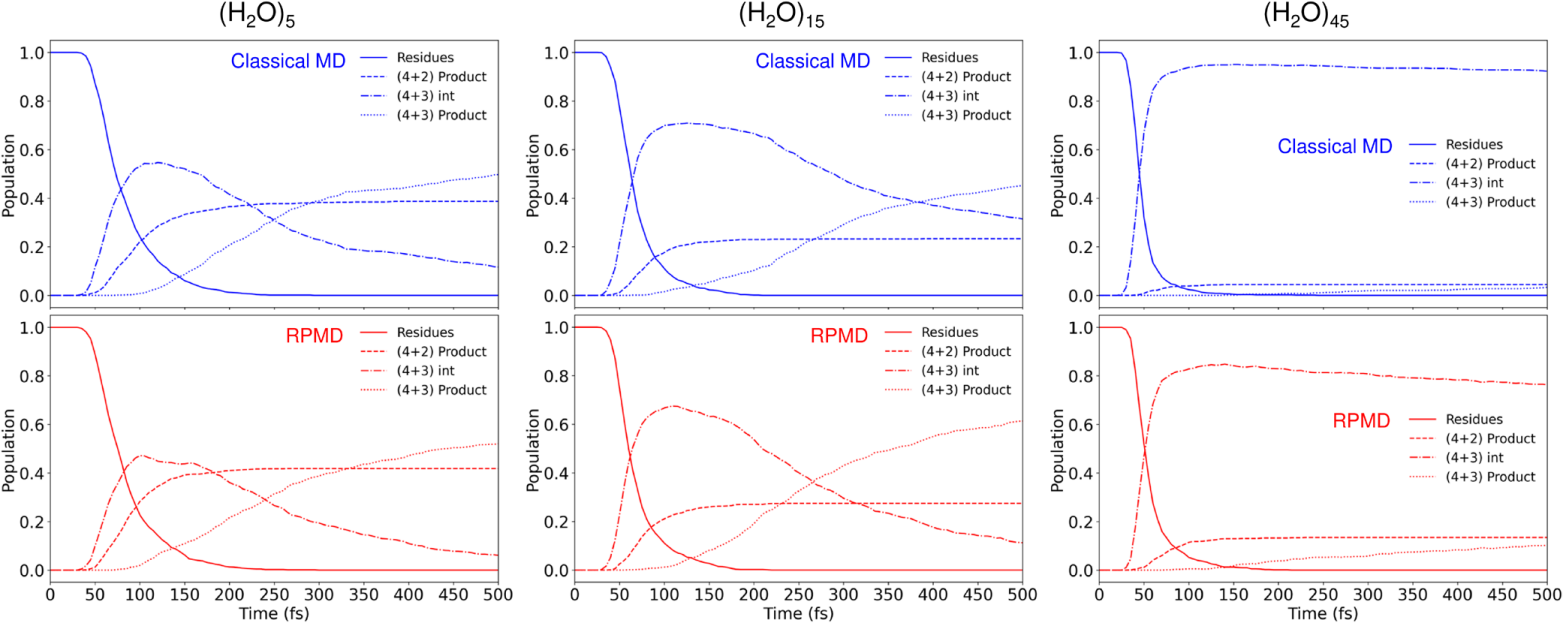
Structural populations as a function of simulation time in terms of 1,3‐butadiene +2‐aminoacrolein reaction obtained from classical MD (upper panels) and RPMD (lower panels) simulation for (H_2_O)_5_ (left), (H_2_O)_15_ (middle), and (H_2_O)_45_ (right) clusters. The dashed, dotted, dash‐dotted, and solid lines represent the populations of (4 + 2) product, (4 + 3) product, (4 + 3) intermediate, and residues, respectively.

The time‐dependent structural populations are presented in Figure 5. In this analysis, we distinguish the (4 + 3) intermediate species from the corresponding (4 + 3) final product in order to compare the ring‐formation times of the (4 + 3) intermediate and the (4 + 2) product. For all cluster sizes, the (4 + 3) intermediate forms more rapidly than the (4 + 2) product as expected from the previous results [43]. Interestingly, for both classical MD and RPMD simulations, the population of the (4 + 3) intermediate initially increases to a maximum and subsequently decreases, while the population of the (4 + 3) product increases correspondingly, indicating the proton‐transfer step that converts the intermediate into the final product. Moreover, the decay of the (4 + 3) intermediate occurs faster in RPMD than in classical MD for all cluster cases. Table 3 summarizes the fractions of proton‐transfer events from the intermediate to the product at the final time step for all cluster sizes considered. In all cluster cases, proton‐transfer events occur more frequently in RPMD than in classical MD, indicating that nuclear quantum effects on the proton motion, even at *T* = 300 K, play a significant role in the proton‐transfer process.

**TABLE 3:** Fractions of proton‐transfer events from the (4 + 3) intermediate to the (4 + 3) product obtained from classical MD and RPMD simulations for the (H_2_O)_5_, (H_2_O)_15_, and (H_2_O)_45_ cluster systems.

	Classical MD	RPMD
(H_2_O)_5_	0.810 ± 0.017 (447/552)	0.895 ± 0.013 (486/543)
(H_2_O)_15_	0.590 ± 0.018 (429/727)	0.845 ± 0.014 (588/696)
(H_2_O)_45_	0.035 ± 0.006 (33/948)	0.118 ± 0.012 (101/853)

*Note:* The numbers in parentheses denote (*N*_prod_/(*N*_int_ + *N*_prod_)), where *N*_prod_ and *N*_int_ represent the number of the (4 + 3) product and the number of (4 + 3) intermediate, respectively. Each fraction is given with its 68% confidence interval, estimated from binomial counting statistics as $\sqrt{p\left(1-p\right)/N}$, where p is the fraction and $N=N_{\mathrm{int}}+N_{\text{prod}}$ is the number of trajectories that formed a product.

**FIGURE 6:**
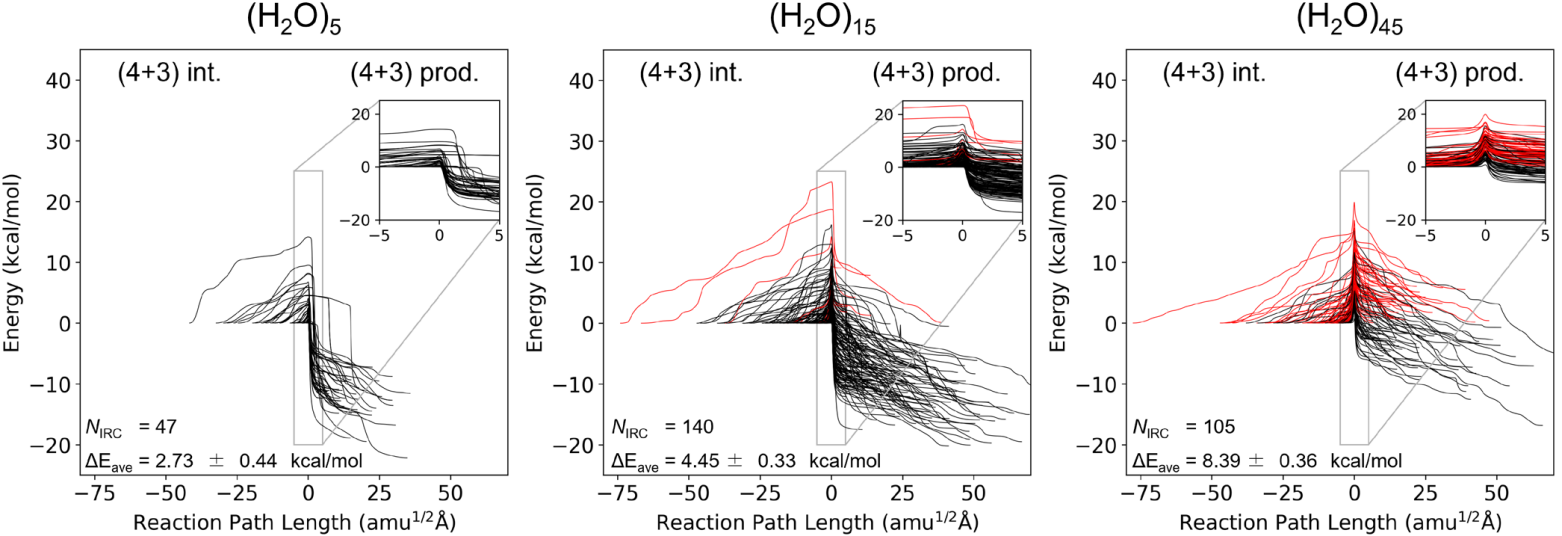
Representative IRC profiles describing the transformation from the (4 + 3) intermediate to the (4 + 3) final product for (H_2_O)_5_ (left), (H_2_O)_15_ (middle), and (H_2_O)_45_ (right) clusters. For each IRC, the zero of energy is defined at the (4 + 3) intermediate. The average barrier heights, together with their standard errors, from the equilibrium structures of the (4 + 3) intermediate to the corresponding transition states are provided in each panel. The numbers of IRCs are 47, 140, 105 for (H_2_O)_5_, (H_2_O)_15_, and (H_2_O)_45_ cluster systems, respectively. The black and red lines correspond to the exothermic and endothermic processes, respectively. The inset panels display enlarged views of the IRC region between reaction path lengths of −5 and 5.

Furthermore, Figure 5 and Table 3 clearly show that the proton‐transfer process becomes significantly suppressed as the number of water molecules increases. This suppression is robust with respect to the propagation time. For the (H_2_O)_45_ cluster, the (4 + 3) intermediate population becomes essentially stationary within ~100 fs, so its small proton‐transfer fraction reflects the high effective barrier and the strong stabilization of the zwitterionic intermediate (Figure 6) rather than insufficient simulation time. For the smaller (H_2_O)_5_ and (H_2_O)_15_ clusters, by contrast, the intermediate‐to‐product conversion is still in progress at 500 fs, so the corresponding fractions in Table 3 are finite‐time populations; nevertheless, the more frequent proton transfer in RPMD than in classical MD is already evident, so the conclusions do not depend on the chosen propagation time. Figure 6 shows the representative IRC profiles for each cluster, describing the conversion from the (4 + 3) intermediate to the (4 + 3) final product. Notice that different IRC profiles come from different water solvation structures, where the initial solvation structure was randomly chosen in these IRC calculations. The average barrier heights from the equilibrium structures of the (4 + 3) intermediate to the corresponding transition states are 2.73, 4.45, and 8.39 kcal/mol for the (H_2_O)_5_, (H_2_O)_15_, and (H_2_O)_45_ cluster systems, respectively. In addition, the red lines shown in Figure 6 represent that the conversion from the zwitterionic (4 + 3) intermediate to the neutral (4 + 3) product is endothermic, with the numbers of red lines being 0, 7, and 50 for the (H_2_O)_5_, (H_2_O)_15_, and (H_2_O)_45_ cluster systems, respectively. Consequently, as the number of polar H_2_O molecules increases, the zwitterionic intermediate is preferentially stabilized relative to the neutral product, thereby raising the effective barrier for the proton‐transfer process. The stabilization is consistent with the well‐known neutral–zwitterion equilibria observed in amino acids [53, 54]. We emphasize that these IRC profiles are static, one‐dimensional, zero‐temperature pathways obtained from a finite set of randomly chosen solvation structures (47, 140, and 105 IRCs for the (H_2_O)_5_, (H_2_O)_15_, and (H_2_O)_45_ clusters, respectively). They are used here only to rationalize the trends qualitatively and do not, by themselves, capture the full finite‐temperature dynamical ensemble; the trend they reveal is nevertheless consistent with the independently computed dynamical proton‐transfer fractions in Table 3.

**FIGURE 7:**
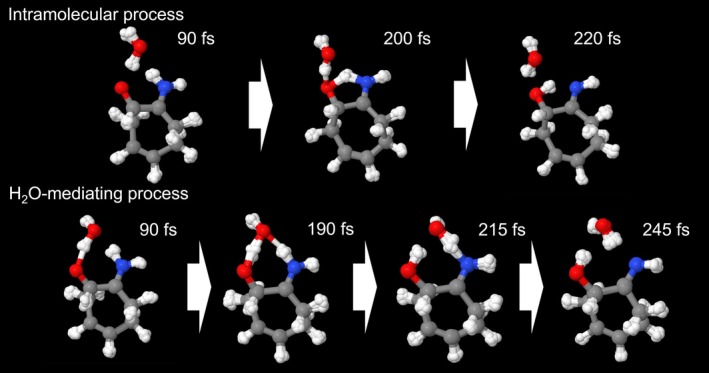
Representative snapshots showing the direct intramolecular proton‐transfer pathway (upper panel) and the indirect H_2_O‐mediated mechanism (lower panel) observed in the RPMD simulations for the (H_2_O)_5_ cluster system.

For the proton‐transfer processes from the zwitterionic (4 + 3) intermediate to the neutral (4 + 3) product, proton migration is considered to occur through both direct intramolecular transfer and water‐mediated shuttling within the water solvent network [55]. Figure 7 presents representative snapshots illustrating both the direct intramolecular pathway and the indirect H_2_O‐mediated mechanism observed in the RPMD simulations for the (H_2_O)_5_ cluster system. As expected, a transient H_3_O^+^ species, appearing at 190 fs, is involved in the H_2_O‐mediated pathway. Furthermore, in our cluster systems, the water‐mediated proton‐transfer process involves a single water molecule acting as the proton shuttle. Table 4 presents the fractions of direct proton‐transfer events among all proton‐transfer processes at the final time step, distinguishing direct intramolecular transfers from water‐mediated pathways for all cluster sizes. For all cluster sizes, the fraction of the direct pathway is higher in classical MD than in RPMD, indicating that the water‐mediated proton‐shuttling pathways are enhanced, likely due to proton tunneling within the hydrogen‐bond network. Interestingly, in the (H_2_O)_45_ cluster system, the RPMD results show that the indirect pathway becomes predominant, suggesting that the water‐mediated mechanism may dominate under bulk‐like aqueous conditions. It should be emphasized that, in theoretical simulations, this mechanism is difficult to capture using implicit solvation models and classical nuclear dynamics approaches.

**TABLE 4:** Fractions of direct proton‐transfer events among all proton‐transfer processes obtained from classical MD and RPMD simulations for the (H_2_O)_5_, (H_2_O)_15_, and (H_2_O)_45_ cluster systems.

	Classical MD	RPMD
(H_2_O)_5_	0.752 ± 0.020 (336/447)	0.671 ± 0.021 (326/486)
(H_2_O)_15_	0.900 ± 0.014 (386/429)	0.582 ± 0.020 (342/588)
(H_2_O)_45_	0.818 ± 0.067 (27/33)	0.198 ± 0.040 (20/101)

*Note:* The numbers in parentheses denote (*N*_dir_/*N*_prod_), where *N*_dir_ represents the number of (4 + 3) products formed via direct proton transfer and *N*_prod_ represents the total number of (4 + 3) products. Each fraction is given with its 68% confidence interval, estimated from binomial counting statistics as $\sqrt{p\left(1-p\right)/N}$, where p is the fraction and $N=N_{\text{prod}}$ is the number of trajectories that formed a product.

## 4 Conclusion

We conducted a theoretical study of the cyclization reaction between 2‐aminoacrolein and 1,3‐butadiene employing an explicit microsolvated model with three water‐cluster sizes—(H_2_O)_5_, (H_2_O)_15_, and (H_2_O)_45_—to investigate the branching dynamics leading to the (4 + 2) and (4 + 3) cyclic products. The initial configurations of the surrounding water molecules around the ambimodal TS differ slightly between the classical and PIMD sampling schemes. For the oxygen atom (O_s_) of 2‐aminoacrolein, the hydrogen atom of the water molecule in the small cluster exhibits stronger interactions due to enhanced quantum fluctuations, whereas this effect is reduced in larger clusters, where the more bulk‐like solvation structure surrounding the O_s_ becomes more diffuse under the influence of NQEs.

The branching fractions between the (4 + 2) and (4 + 3) cyclic species show an increasing contribution of the (4 + 3) product channel with larger clusters, consistent with classical MD trends; however, RPMD predicts 3%–9% lower (4 + 3) fraction than classical MD. The inclusion of ZPE can promote the formation of products that deviate from the dominant IRC pathway. This shift in the branching fraction is a direct consequence of NQEs acting on the bifurcation step and demonstrates that an accurate description of the branching behavior requires the explicit inclusion of NQEs. Time‐dependent population analysis shows faster decay of the zwitterionic (4 + 3) intermediate in RPMD than in classical MD, indicating that nuclear quantum effects on the proton motion, even at *T* = 300 K, significantly contribute to the proton‐transfer process. At the same time, proton transfer becomes less favorable as the cluster size increases, suggesting that polar water molecules preferentially stabilize the zwitterionic (4 + 3) intermediate relative to the neutral (4 + 3) product, thereby raising the effective barrier for proton transfer. Remarkably, the indirect water‐mediated proton‐transfer pathway becomes dominant only in RPMD for the (H_2_O)_45_ cluster, suggesting that the water‐mediated mechanism may prevail under bulk‐like aqueous conditions. It should be emphasized that such a mechanism is difficult to capture in theoretical studies employing implicit solvation models and classical nuclear dynamics. The insights obtained in this study provide a mechanistic explanation for the cluster‐size dependence of branching behavior and highlight that reliable modeling of both the bifurcation branching and the subsequent proton‐transfer processes in microsolvated environments requires explicit consideration of both solvation structure and NQEs.

## Author Contributions

**Shoto Nakagawa:** data curation, investigation, visualization, writing – original draft. **Hayato Matsubuchi:** data curation, investigation, visualization. **Haruki Ota:** data curation, investigation, visualization. **Toshiyuki Takayanagi:** conceptualization, funding acquisition, investigation, project administration, supervision, writing – review and editing. **Tatsuhiro Murakami:** conceptualization, funding acquisition, investigation, methodology, project administration, supervision, writing – review and editing.

## Funding

This work was supported by Japan Society for the Promotion of Science London (JP25K08573) and Institute for Quantum Chemical Exploration (R05Josei005, R07Josei005).

## Conflicts of Interest

The authors declare no conflicts of interest.

## Supporting information


**Data S1:** jcc70458‐sup‐0001‐Supinfo.pdf.
**Figure S1:** Validation of the parameter‐optimized GFN2‐xTB potential against B3LYP‐D3/6‐31+G(d,p) for the (H2O)15 cluster, resolved into the four reaction regions: (i) the ambimodal TS region, (ii) the (4 + 2)/(4 + 3) branching region, (iii) the zwitterionic (4 + 3) intermediate region, and (iv) the proton‐transfer barrier region. Each panel shows a representative IRC. The energy profiles at the B3LYP‐D3/6‐31+G(d,p) (black solid), parameter‐optimized GFN2‐xTB (red dashed), and default GFN2‐xTB (blue dotted) levels are depicted. The transition state lies at reaction‐path length zero and is named at the top of each panel (the ambimodal TS for (i) and (ii), the Cope TS for (iii), and the proton‐transfer TS for (iv)); the species at the two ends of the path are labeled at the lower corners. The mean optimized‐vs‐DFT RMSD over all IRCs in the region is indicated in each panel.
**Figure S2:** All 17 IRCs of the (H2O)15 cluster for the same four reaction regions.
**Table S1:** The bond distances R1, R2, and R3 (Å) at the ambimodal transition state of the (H2O)15 cluster optimized using parameter‐optimized GFN2‐xTB, evaluated on the five IRC structures. Values are mean ± standard error over the five structures. The gas‐phase reference values, which were used to seed the dynamics, are also shown for comparison.

## Data Availability

The data that support the findings of this study are available from the corresponding author upon reasonable request.
