# Supplementary material for: Nuclear Quantum Effects on the Organic Bifurcation Reaction in Microsolvated Water Clusters: Ring‐Polymer Molecular Dynamics Calculations Using an Explicit Solvation Model
Source: J Comput Chem. 2026 Jul 6;47(19):e70458. doi: 10.1002/jcc.70458 (PMC13338448; doi:10.1002/jcc.70458)
Supplement: Supplementary file 1 — Data S1: jcc70458‐sup‐0001‐Supinfo.pdf. Figure S1: Validation of the parameter‐optimized GFN2‐xTB potential against B3LYP‐D3/6‐31+G(d,p) for the (H2O)15 cluster, resolved into the four reaction regions: (i) the ambimodal TS region, (ii) the (4 + 2)/(4 + 3) branching region, (iii) the zwitterionic (4 + 3) intermediate region, and (iv) the proton‐transfer barrier region. Each panel shows a representative IRC. The energy profiles at the B3LYP‐D3/6‐31+G(d,p) (black solid), parameter‐optimized GFN2‐xTB (red dashed), and default GFN2‐xTB (blue dotted) levels are depicted. The transition state lies at reaction‐path length zero and is named at the top of each panel (the ambimodal TS for (i) and (ii), the Cope TS for (iii), and the proton‐transfer TS for (iv)); the species at the two ends of the path are labeled at the lower corners. The mean optimized‐vs‐DFT RMSD over all IRCs in the region is indicated in each panel. Figure S2: All 17 IRCs of the (H2O)15 cluster for the same four reaction regions. Table S1: The bond distances R1, R2, and R3 (Å) at the ambimodal transition state of the (H2O)15 cluster optimized using parameter‐optimized GFN2‐xTB, evaluated on the five IRC structures. Values are mean ± standard error over the five structures. The gas‐phase reference values, which were used to seed the dynamics, are also shown for comparison. [file JCC-47-0-s001.pdf]

Supporting Information

Nuclear Quantum Effects on the Organic Bifurcation Reaction in Microsolvated Water Clusters: Ring-Polymer Molecular Dynamics Calculations Using an Explicit Solvation Model

Shoto Nakagawa[1], Hayato Matsubuchi[1], Haruki Ota[1], Toshiyuki Takayanagi[1], and Tatsuhiro Murakami[*2]

[1]Department of Chemistry, Saitama University, Shimo-Okubo 255, Sakura-ku, Saitama 338-8570, Japan
[2]Department of Applied Chemistry for Environment, Graduate School of Urban Environmental Sciences, Tokyo Metropolitan University, 1-1 Minami-Osawa, Hachioji-shi, Tokyo 192-0397, Japan

Contents

S1 Benchmarking of the parameter-optimized GFN2-xTB potential **S2**

S2 Definition of the first-shell hydration number **S3**

[*]Corresponding author. E-mail: murakamit@tmu.ac.jp, ORCID: 0000-0001-8904-8673

S1 Benchmarking of the parameter-optimized GFN2-xTB potential

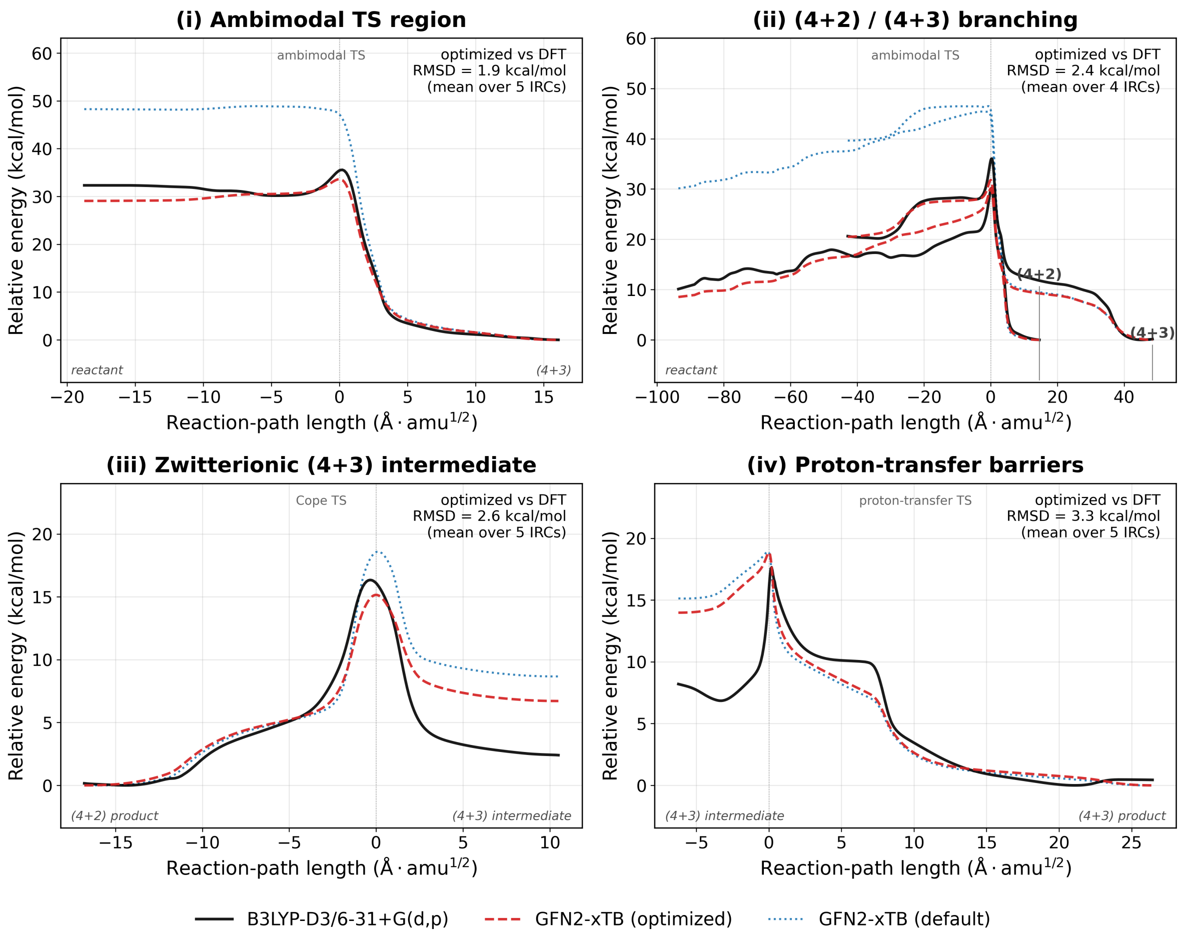

Figure S1: Validation of the parameter-optimized GFN2-xTB potential against B3LYP-D3/6-31+G(d,p) for the $(H_2O)_{15}$ cluster, resolved into the four reaction regions: (i) the ambimodal TS region, (ii) the (4+2)/(4+3) branching region, (iii) the zwitterionic (4+3) intermediate region, and (iv) the proton-transfer barrier region. Each panel shows a representative IRC. The energy profiles at the B3LYP-D3/6-31+G(d,p) (black solid), parameter-optimized GFN2-xTB (red dashed), and default GFN2-xTB (blue dotted) levels are depicted. The transition state lies at reaction-path length zero and is named at the top of each panel (the ambimodal TS for (i) and (ii), the Cope TS for (iii), and the proton-transfer TS for (iv)); the species at the two ends of the path are labeled at the lower corners. The mean optimized-vs-DFT RMSD over all IRCs in the region is indicated in each panel.

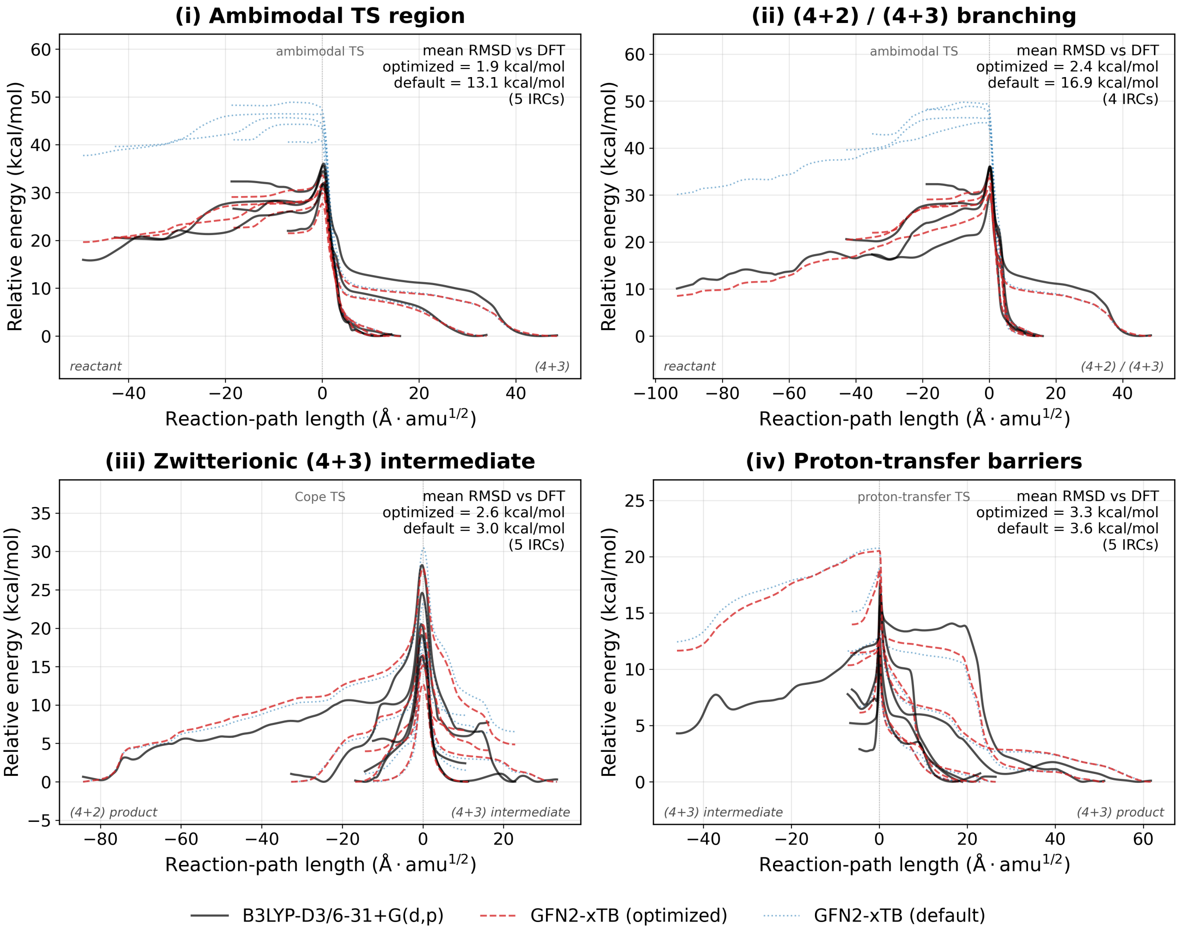

Figure S2: All 17 IRCs of the $(H_2O)_{15}$ cluster for the same four reaction regions

Table S1: The bond distances R_1, R_2, and R_3 (Å) at the ambimodal transition state of the $(H_2O)_{15}$ cluster optimised using parameter-optimized GFN2-xTB, evaluated on the five IRC structures. Values are mean ± standard error over the five structures. The gas-phase reference values, which were used to seed the dynamics, are also shown for comparison.

	$(H_2O)_{15}$ ambimodal TS	Gas phase
R_1	2.25 ± 0.02	2.06
R_2	2.91 ± 0.01	2.81
R_3	2.57 ± 0.02	2.59

S2 Definition of the first-shell hydration number

The average number of water hydrogen atoms in the first solvation shell around the carbonyl oxygen (O_s) of 2-aminoacrolein, $\bar{n}_H$ (Table 1 and Figure 3 of the main

text), is defined as the running coordination number of the O_s–H pair distribution, evaluated up to the first minimum R_c of the radial distribution function (RDF):

$$\bar{n}_H = \left\langle \sum_{k=1}^{N_H} \theta(R_c - r_{O_s H_k}) \right\rangle_{\text{conf, bead}} = \rho \int_0^{R_c} 4\pi r^2 g_{O_s H}(r)\, dr, \tag{S1}$$

where $r_{O_s H_k}$ is the distance between O_s and the k-th water hydrogen atom, $N_H = 2n$ is the total number of water hydrogen atoms in the $(H_2O)_n$ cluster, and θ is the Heaviside step function. The angle brackets $\langle \cdot \rangle_{\text{conf, bead}}$ denote an average over the M sampled configurations ("conf") and, for the PIMD results, additionally over the $P = 32$ ring-polymer beads ("bead"; $P = 1$ recovers the classical average); ρ is the number density of water and R_c is the first minimum of $g_{O_s H}(r)$. With this normalization $\bar{n}_H$ is the quantum-mechanical expectation value of the number of water hydrogen atoms within R_c of O_s.

The O_s–H radial distribution function $g_{O_s H}(r)$ is obtained from the sampled O_s–H distances. For classical MD each hydrogen contributes a single distance per configuration,

$$g^{\text{cl}}_{O_s H}(r) = \frac{1}{\rho\, 4\pi r^2} \frac{1}{M} \sum_{m=1}^{M} \sum_{k=1}^{N_H} \delta\left(r - r^{(m)}_{O_s H_k}\right), \qquad r^{(m)}_{O_s H_k} = \left|\mathbf{r}^{(m)}_{H_k} - \mathbf{r}^{(m)}_{O_s}\right|, \tag{S2}$$

where $m = 1, \ldots, M$ labels the sampled configurations. For PIMD (and the RPMD sampling, which preserves the same quantum Boltzmann distribution) every nucleus is represented by a ring polymer of P beads, and the distribution is obtained by averaging the same-bead (same imaginary-time slice) distances over all P beads,

$$g^{\text{PIMD}}_{O_s H}(r) = \frac{1}{\rho\, 4\pi r^2} \frac{1}{M} \sum_{m=1}^{M} \frac{1}{P} \sum_{s=1}^{P} \sum_{k=1}^{N_H} \delta\left(r - r^{(m,s)}_{O_s H_k}\right), \qquad r^{(m,s)}_{O_s H_k} = \left|\mathbf{q}^{(m,s)}_{H_k} - \mathbf{q}^{(m,s)}_{O_s}\right|, \tag{S3}$$

where $\mathbf{q}^{(m,s)}_X$ is the position of bead s of nucleus X in configuration m and $P = 32$; setting $P = 1$ recovers Eq. (S2). Consequently, the first-shell count $\bar{n}_H$ is obtained

directly as

$$\bar{n}_{\mathrm{H}} = \frac{1}{MP} \sum_{m=1}^{M} \sum_{s=1}^{P} \sum_{k=1}^{N_{\mathrm{H}}} \theta \left(R_{\mathrm{c}} - r_{\mathrm{O_s}H_k}^{(m,s)} \right). \qquad \text{(S4)}$$

The cutoff was set to $R_{\mathrm{c}} = 2.6$ Å and applied identically to the classical MD and PIMD analyses and to all cluster sizes.
